# Supplementary figures and images for: Landscape and female fertility evaluation of seven heavenly bamboo cultivars as potential non-invasive alternatives to the wildtype
Source: PLoS One. 2024 Sep 20;19(9):e0310246. doi: 10.1371/journal.pone.0310246 (PMC11414976; doi:10.1371/journal.pone.0310246)

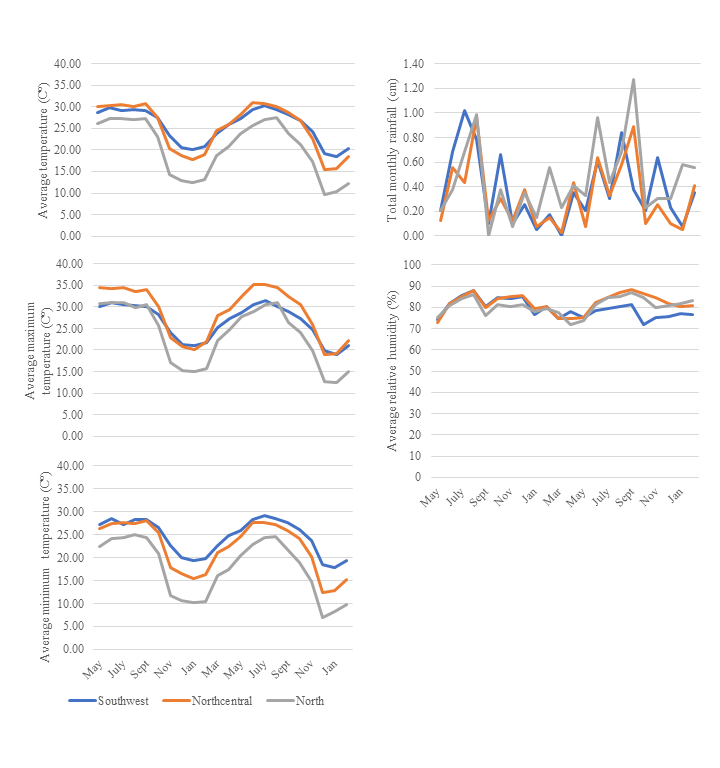

Supplement: S1 Fig — Sites located at southwest FL [Gulf Coast Research and Education Center (GCREC), Balm], northcentral FL [Plant Science Research and Education Unit (PSREU), Citra], and north FL [North Florida Research and Education Center (NFREC), Quincy]. Where week 0 starts in May 2019 and Week 82 ends in January 2020. (TIF) [file pone.0310246.s001.tif]

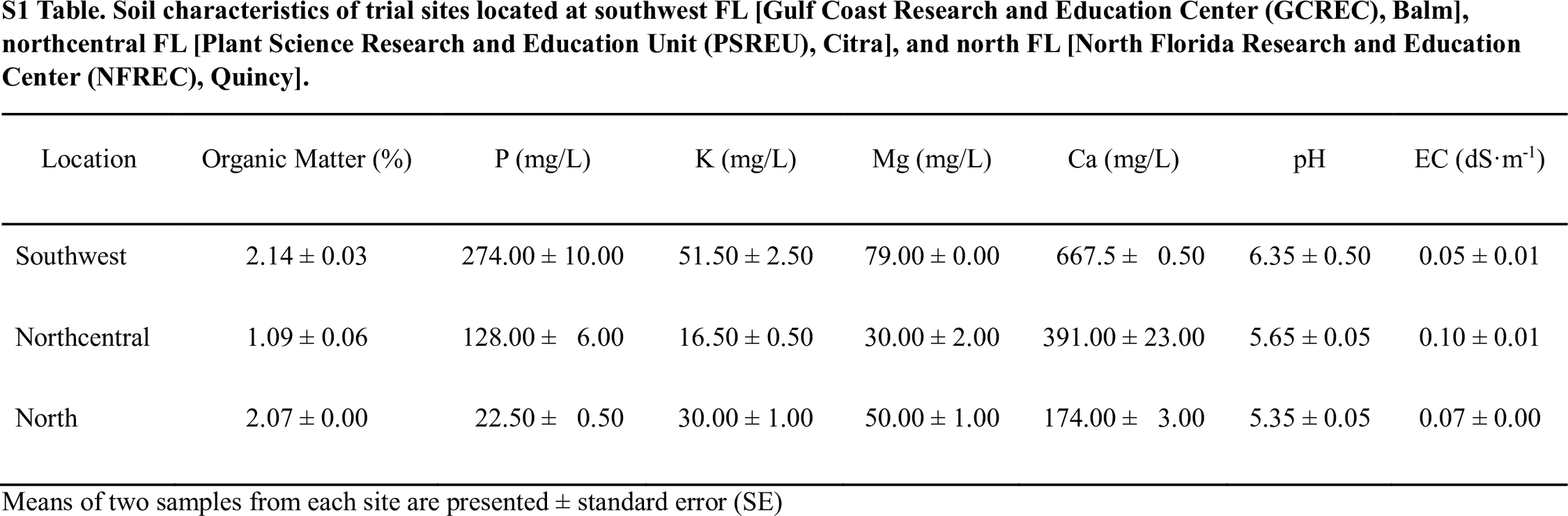

Supplement: S1 Table — (TIF) [file pone.0310246.s002.tif]
